# How inclusive are cell lines in preclinical engineered cancer models?

**DOI:** 10.1242/dmm.049520

**Published:** 2022-06-01

**Authors:** Shreya Raghavan

**Affiliations:** Department of Biomedical Engineering, Texas A&M University, 3120 TAMU, 5016 Emerging Technologies Building, College Station, TX 77843, USA

## Abstract

Diverse factors contribute to significant and dire disparities in cancer risk and treatment outcomes. To address this, there was a call for inclusion of sex as a biological variable, which resulted in more instances of careful inclusion of sex in preclinical studies of cancer. Another variable in cancer treatment is genetic ancestry. Although this is considered explicitly in clinical research, it is considerably neglected in preclinical studies. Preclinical research can use several 3D *in vitro* model systems, such as spheroids/organoids, xenografts, or other bioengineered systems that combine biomaterials and cellular material. Ultimately, the cellular base for all of these *in vitro* model systems is derived from human cell lines or patient samples, to investigate mechanisms of cancer and screen novel therapeutics, all of which aim to maximize successful outcomes in clinical trials. This in itself offers an opportunity to potentiate effective treatments for many groups of people, when diverse variables like genetic ancestry are consciously included into study design. This Perspective highlights the need for conscious inclusion of genetic ancestry in preclinical cancer tissue engineering, especially when it pertains to determining therapeutic outcomes.

## Abstract (precis)

**Summary:** Genetic determinants, like ancestry, impact cancer risk and therapeutic outcomes. Hence, this is an important variable to consider at the very initial stages of biomedical research at the bench.

## Powerful opportunities in preclinical cancer models

Advances in regenerative engineering technologies have resulted in the expansive adoption of *in vitro* preclinical disease models in biomedical research. Several types of *in vitro* models are used widely in cancer research, including spheroids/organoids (Drost and Clevers, 2018), xenografts (Olson et al., 2018; Hidalgo et al., 2014) and other nuanced models that combine biomaterial scaffolding with cellular material (Marturano-Kruik et al., 2016). Spheroids/organoids combine aggregates of single or multiple cell types within a 3D sphere-like environment. Xenografts are tumors generated predominantly in rodents and are used to bridge *in vitro* and *in vivo* mechanisms. Other nuanced approaches to create bioengineered *in vitro* models exist, including organ-on-chip approaches and diverse biomaterial scaffolding strategies. At their core, all of these models transform cellular material, either from established immortalized cell lines or patient-derived cells, into 3D tissues that can mimic various aspects of *in vivo* tumors.

**Fig. 1.**
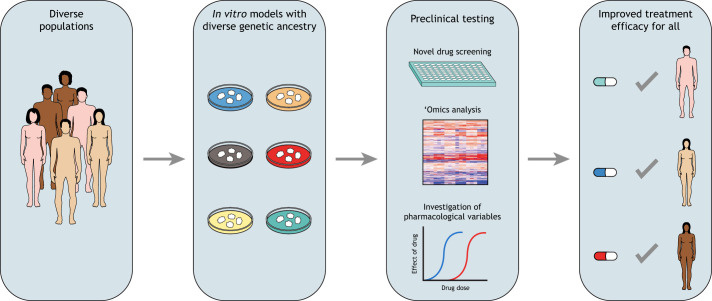
**The promise of preclinical research that consciously includes genetic ancestry as a variable is that precision medicine becomes a reality for all populations of people, and not just a majority.** By expanding the genetic diversity of *in vitro* models in preclinical testing, we can improve clinical trial outcomes and treatment efficacy for all.

These models have various uses, from being able to study and modulate cell and tissue behaviors to evaluating responses to therapeutics in a preclinical setting. The field of cancer extensively utilizes preclinical models to identify tumor dependencies and modalities that can be targeted by novel therapeutics. In this context, 3D *in vitro* models have made significant strides in the past three decades, presenting substantial advantages over monolayer cell cultures (Pinto et al., 2020; Brancato et al., 2020). Notably, the architectural and structural complexity and superior cell–cell interactions promote diffusional limitations that mimic *in vivo* interactions, without the need to employ animal models in the early stages of a study. Importantly, 3D *in vitro* models offer less variability and are often more cost effective than the more laborious animal model studies (Day et al., 2015). One example of the wide adoption of 3D models is the spheroid/organoid models used extensively in preclinical chemotherapy screens in the oncology research space (Raghavan et al., 2017). However, there are gaps in the considerations made while building such *in vitro* preclinical models, as further action is needed to promote the development of more inclusive *in vitro* cancer models that represent the populations affected by these diseases. An obvious question to ask at this point is why this conscious inclusion matters (Fig. 1).

Well-documented disparities exist in cancer incidence and risk, treatment outcomes and survival across diverse populations (DeSantis et al., 2016; Torre et al., 2016). Lifestyle and socioeconomic factors are central to the reported incidence disparity (Singh and Jemal, 2017; Heitz et al., 2018; Wilson and Mucci, 2019; Brookman-May et al., 2019), and sex as a biological variable has gained increasing attention. However, the role of genetic ancestry remains largely neglected at some stages of clinical research and virtually all stages of preclinical cancer research, and even biomedical research at large. For example, compared with other ethnic groups in the United States, Black Americans have higher rates of incidence of colorectal cancer, yet are under-represented and under-enrolled in clinical trials for colorectal cancer (Augustus and Ellis, 2018; Awidi and Al Hadidi, 2021).

Extensive preclinical research has the power to determine the potential success of therapeutic intervention and identify specific cohorts of patient populations for whom it is likely to be most beneficial for, but this is only possible when it is inclusive. As preclinical study outcomes ultimately determine who is eligible to participate in a clinical study, a systematic review of preclinical studies was undertaken to guide the inclusion criteria for clinical trials (Sena et al., 2014). Inclusion criteria are designed to minimize the impact of confounding variables on clinical study outcomes (Keung et al., 2020). Clinical cancer trials often have narrow inclusion criteria, such as inclusion of patients with specific molecular signatures  (Fakih et al., 2022) or exclusion of individuals who previously received chemotherapy (Pruitt et al., 2017), to maximize their success and validity (Britton et al., 1999).

Therefore, when genetic ancestry is not explicitly considered in *in vitro* preclinical research, researchers are simply not developing treatments that work for all groups of people. This overt lack of inclusivity exacerbates disparity in treatment outcomes. A recent article surveyed the biomedical research landscape in regenerative engineering to conclude that ancestral background was significantly under-reported in preclinical *in vitro* studies (Ryan et al., 2021). We aim to show here that the under-representation of ethnically diverse enrollees in clinical cancer trials (Aldrighetti et al., 2021) can in part be improved with expansive and inclusive preclinical cancer research. This Perspective will highlight current limitations in preclinical cancer tissue engineering in the context of inclusivity and provide thoughts on future actions to tackle this issue.

“Conscious inclusion of genetic ancestry in *in vitro* models will enable the identification of the root cause of disparity in treatment, pharmacological variables and even tumor biology. If we continue to ignore this variable as an inconvenience, we are willfully contributing to a system of perpetuating inequity.”

## Lessons learned from the ‘sex as a biological variable’ movement

An emerging body of evidence in the past decade suggested that sex is a powerful biological variable that affects several outcomes, including cancer incidence, response to (chemo)therapy and mortality. Even in cancers that are not related to sex organs, there are significant disparities in chemotherapy outcomes. For example, response to chemotherapy is more favorable in female compared to male melanoma patients (Rampen, 1982). Furthermore, 5-fluorouracil, a commonly used chemotherapeutic agent for gastrointestinal malignancies, is eliminated from males at higher rates than from females, leading to different pharmacokinetics and thereby a difference in drug effects (Mueller et al., 2013). Therefore, it is advantageous to incorporate sex as a significant biological variable at early stages of disease model building by powering *in vitro* studies with as many female as there are male samples. This could lead to robust new strategies that identify the biological basis of sex differences in cancer (Wagner, 2020). Importantly, sex-specific dose modifications, or other sex-specific strategies that improve treatment efficacy, could emerge from careful inclusion of sex as a biological variable in *in vitro* disease models. Similarly to sex, other genetically determined factors inevitably influence cancer risk and response to treatment, and warrant further consideration.

## The promise and shortfall of cell lines to diversify genetic ancestry in preclinical research

**Fig. 2.**
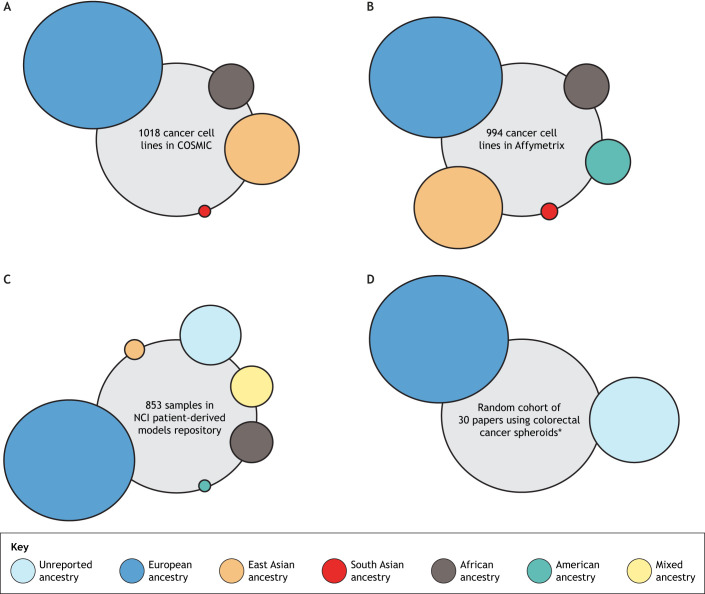
**Reported diversity of patient samples and cell lines available through common databases and repositories.** (A) Of 1018 cancer cell lines in the Catalogue of Somatic Mutations in Cancer (COSMIC), 317 had reported ancestry and 701 had unreported ancestry; 697 of these cell lines were of European ancestry (245 reported and 453 unreported), 253 were of East Asian ancestry (37 reported and 215 unreported), 56 were of African ancestry (26 unreported and 30 reported), and one cell line of unreported ancestry was found to be South Asian (Kessler et al., 2019). (B) The Affymetrix SNP6.0 arrays genotyping dataset contains 994 cancer cell lines that are deposited in the European Genome-Phenome Archive. Analysis of this dataset found that 633 of the cell lines were of European ancestry, 248 were of East Asian ancestry, 56 were of African ancestry, 47 were of American ancestry and ten were of South Asian ancestry (Nguyen et al., 2021). (C) Of 853 samples in the NCI patient-derived models repository, 135 had unreported ancestry, 645 were of European ancestry, 39 were of African ancestry, 25 were of mixed ancestry, eight were of East Asian ancestry, and one was of American ancestry (NCI-Frederick). (D) A literature review of 30 peer-reviewed manuscripts used in colorectal cancer preclinical research (Table S1); 22 of these studies used established cell lines, all of which were of European ancestry (https://web.expasy.org/cellosaurus), and eight of these studies used patient samples, all of which had unreported ancestry. *Numbers represent studies instead of individual cell lines or samples.

With the advent of personalized and precision medicine, cancer therapeutics are already being tailored based on genetic alterations. For example, melanoma patients with the *BRAF V600E* mutation benefit from improved survival with vemurafenib treatment (Young et al., 2012; Chapman et al., 2011). Such nuances in molecular targeting are foundationally driven by preclinical testing and research in established panels of cancer cell lines with extensive characterization on their genetic alterations, like that in the NCI-60 set established by the National Cancer Institute (NCI). Whether in monolayer cultures or 3D *in vitro* settings, these cell lines allow testing of targeted anti-cancer therapies (Basu et al., 2013; Shoemaker, 2006). The NCI-60 panel of cell lines was vital in standardizing preclinical assays for all stages of the anti-cancer drug discovery pipeline, including the use of regenerative engineering and high-throughput screening. Several other panels, like the Cancer Cell Line Encyclopedia (CCLE) and the Catalogue of Somatic Mutations in Cancer (COSMIC) cell lines project, expanded the number of cell line models available for preclinical cancer research. However, the opportunity to include self-reported and verified genetic ancestry data was missed. Of the 1018 cancer cell lines in the COSMIC, 701 lines had unreported ancestry, of which 453 were inferred to be European (Fig. 2) (Kessler et al., 2019). A similar trend was noted in the CCLE lines as well (Dutil et al., 2019).

The Cancer Genome Atlas (TCGA), a multi ‘omics database, initially propelled the identification of oncogenes and driver mutations in cancer (Cancer Genome Atlas Research Network et al., 2013), but only a small cohort contains self-identified race or ethnicity data. Using these data, however, several groups have shown that the genetic backgrounds of patients influence somatic mutations in oncogenes, biomarker genes and cancer drivers during cancer incidence (Carrot-Zhang et al., 2020). With advances in genome-wide association studies, a Cancer Genetic Ancestry Atlas (TCGAA) has been created to allow the visualization of TCGA data superimposed with the inferred genetic ancestry of patients across 33 different cancer types (Yuan et al., 2018). Inferred ancestry data from cell lines also indicate that genetic ancestry contributes to disparity in chemotherapy outcomes (Jack et al., 2015), providing a clear basis for the inclusion of diverse ethnic samples in preclinical research. Disappointingly, but not surprisingly, analysis of datasets from over 1000 commonly used cancer cell lines demonstrated that 63% of them had European ancestry (Fig. 2) (Nguyen et al., 2021). In an attempt to better recapitulate the genetic heterogeneity and diversity of cancer patients, the NCI moved towards a patient-derived models repository (NCI-Frederick) that includes xenografts, *in vitro* tumor cell cultures and organoids. This was an obvious solution to overcome the limitations of established cell lines, but they still fell short of the goal to expand the diversity of genetic ancestry. Of the samples in this repository, 46.1% had no race/ethnicity data, while the majority of the rest were derived from self-identified White/European patients (Fig. 2). A comprehensive and disappointing breakdown of race/ethnicity of other biobanks and repositories is summarized by Guerrero et al. (2018).

“Does this mean that every study, at every point (pilot, foundational or translational), should compulsorily use diverse cell lines? The answer is likely no, because mandating this will exclude many scientists who simply lack the resources to do so, despite the awareness of conscious inclusion.”

To assess how much of this inclusion problem permeated into cancer spheroid models, I reviewed the literature published from 2017 to present (the past 5 years) using colorectal cancer spheroids. From a random cohort of 30 reviewed papers, 73.4% used established cell lines like HCT-116 and HT-29 in their studies, both of which cluster under white/European ancestry profiles, while only 26.7% used patient-derived cells (Table S1 and Fig. 2). Of the ones that used patient-derived cells, there were no reports of either sex as a biological variable or ancestry/ethnicity. Although cell lines obviously offer a well-characterized molecular template, attempting preclinical research with multiple cell line panels that have greater documented diversity in ancestry is crucial to tackling therapeutic disparity. Conscious inclusion of genetic ancestry in *in vitro* models will enable the identification of the root cause of disparity in treatment, pharmacological variables and even tumor biology. If we continue to ignore this variable as an inconvenience, we are willfully contributing to a system of perpetuating inequity.

To begin, we must acknowledge the problem as it stands today: there are limitations in available cell lines with reported and diverse genetic ancestry (reported above), and there is occasional mis-reporting of this information in commonly used cell lines (Hooker et al., 2019). Significant lack of ethnic diversity in clinical trials permeates to biorepository collections in the United States that are not representative of its own ethnic demographic. Therefore, curating diverse cell lines and patient-derived cells requires global cooperation with significant sample sharing, while acknowledging that significant infrastructural investment is required for biorepository maintenance in low-resource settings (Abimiku et al., 2019; Mendy et al., 2014).

## Where do we go from here?

One obvious strategy to incentivize the inclusion of diverse samples in studies is recognizing and acknowledging the gap and adding it to the ‘Rigor’ criteria of research proposal evaluations. This lies in the hands of large funding agencies, and perhaps even journal publishers. This approach, while essentially forcing researchers' hands, has been widely successful in incorporating sex as a biological variable, and has thus set a precedent for including ancestry information as an equally relevant variable.

A cautionary aspect of forcing inclusion, however, is the lack of formalized definitions for ancestral breakdown. The United States Census-based definition lacks inclusivity (Telles, 2018; Strmic-Pawl et al., 2018) and considers huge bulks of ethnically diverse populations as monoliths. Latinos, for example, are a diverse ethnic category that includes individuals from several different regions, with differences in cancer incidence and risk (Stern et al., 2016). Hence, a meaningful categorization of genetic ancestry-based groups has to be created before mandating the inclusion of ancestry as a variable. I have no doubt that consortia of international scholars can use genetic data-driven practices to establish these categories in an inclusive manner. Initiatives like the 1000 Genomes Project that catalogues human genetic variation across populations (1000 Genomes Project Consortium et al., 2015; Siva, 2008) need wider attention outside of the genetics field by involving social scientists, biologists and bioengineers.

Does this mean that every study, at every point (pilot, foundational or translational), should compulsorily use diverse cell lines? The answer is likely no, because mandating this will exclude many scientists who simply lack the resources to do so, despite the awareness of conscious inclusion. However, researchers who enjoy the privilege of using patient-derived cells/lines should report the known ethnicity of their samples when disseminating scientific data through peer-reviewed publications. The charge and onus to change this lies with bigger institutions, universities and funding agencies like the NCI. Universities that house precision medicine centers and biorepositories could curate ethnically diverse cell lines for broader use by their researchers. If universities can extend their educational reach through global partnerships, they certainly can extend cooperative hands to stock biorepositories that capture ethnic diversity globally. Federal agencies like the National Institutes of Health (NIH) and its institutes leading this charge will have implications not just on cancer research, but on all disease models that benefit from preclinical research.

“If universities can extend their educational reach through global partnerships, they certainly can extend cooperative hands to stock biorepositories that capture ethnic diversity globally.”

Any time a change in our fundamental way of doing things is asked of academics, there is resistance. However, combating health disparity is a collective responsibility borne by everyone at every training level in academic research, and not just medical and health professionals. Being conscious of inclusion during our development of disease models and using the weight of bioengineering to power preclinical studies are sure to make a difference. The aspirational goal of cancer research should be to make a difference for all people. Talking about disparity and inequity is simply not enough anymore, if it does not permeate into action at the academic research level. The time for that action is now.

## Supplementary Material

Supplementary information
