# Supplementary material for: How inclusive are cell lines in preclinical engineered cancer models?
Source: Dis Model Mech. 2022 Jun 1;15(5):dmm049520. doi: 10.1242/dmm.049520 (PMC9187871; doi:10.1242/dmm.049520)
Supplement: Supplementary information [file dmm-15-049520-s1.pdf]

Table S1. Thirty random 'colorectal cancer spheroid' studies from 2017 onwards

Study/ Citation	Cells used	Ancestry known?
(Yusefi et al., 2021)	HCT-116, HT-29	European
(Lee et al., 2019)	HCT-116	European
(Silva et al., 2018)	HT-29	European
(Rengganaten et al., 2020)	HC-15, WiDr (HT-29 derivative)	European
(Tomeh et al., 2021)	HCT-116	European
(Khot et al., 2018)	HT-29, HCT-116	European
(Jeppesen et al., 2017)	22 patient samples	Unreported
(Kasper et al., 2020)	HT-29, HCT-116	European
(Árnadóttir et al., 2018)	6 patient samples	Unreported
(Pereira et al., 2019)	HT-29	European
(Cattin et al., 2018)	HCT-116, SW620	European
(Cianciosi et al., 2020)	HCT-116	European
(Rousset et al., 2022)	HCT-116	European
(Hachey et al., 2021)	HCT-116, SW480	European
(Serna et al., 2020)	Patient samples	Unreported
(Griseti et al., 2019)	HCT-116	European
(Thakuri et al., 2019)	HCT-116	European
(Ranjbar-Mohammadi et al., 2019)	HT-29	European
(Courau et al., 2019)	HT-29	European
(Agarwal et al., 2019)	HT-29, SW480	European
(Lamberti et al., 2019)	SW480	European
(Baranyi et al., 2020)	HCT-116, DLD-1, SW480, WiDr (HT-29 derivative)	European
(Miyoshi et al., 2018)	141 patient samples	Unreported
(Di Mascolo et al., 2019)	26 patient samples	Unreported
(Xu et al., 2019)	SW480, HCT-116	European
(Agarwal et al., 2020)	116 patient samples	Unreported
(Weber et al., 2019)	2 patient samples	Unreported
(Sargenti et al., 2020)	HT-29, SW620,	European
(Turano et al., 2018)	Patient samples	Unreported
(Ruiz et al., 2022)	HT-29	European
